# Contrastive Numerical Investigations on Thermo-Structural Behaviors in Mass Concrete with Various Cements

**DOI:** 10.3390/ma9050378

**Published:** 2016-05-20

**Authors:** Wei Zhou, Chuqiao Feng, Xinghong Liu, Shuhua Liu, Chao Zhang, Wei Yuan

**Affiliations:** 1 State Key Laboratory of Water Resources and Hydropower Engineering Science, Wuhan University, Wuhan 430072, Hubei, China; zw_mxx@163.com (W.Z.); fcq0418@163.com (C.F.); shliu@whu.edu.cn (S.L.); yw_0201@126.com (W.Y.); 2 Changjiang Survey Planning Design & Research Company Limited, Wuhan 430010, Hubei, China; gonopo2006@126.com

**Keywords:** early-age, supersulfated cement concrete, hydration model, multi-scale

## Abstract

This work is a contrastive investigation of numerical simulations to improve the comprehension of thermo-structural coupled phenomena of mass concrete structures during construction. The finite element (FE) analysis of thermo-structural behaviors is used to investigate the applicability of supersulfated cement (SSC) in mass concrete structures. A multi-scale framework based on a homogenization scheme is adopted in the parameter studies to describe the nonlinear concrete behaviors. Based on the experimental data of hydration heat evolution rate and quantity of SSC and fly ash Portland cement, the hydration properties of various cements are studied. Simulations are run on a concrete dam section with a conventional method and a chemo-thermo-mechanical coupled method. The results show that SSC is more suitable for mass concrete structures from the standpoint of temperature control and crack prevention.

## 1. Introduction

Cracking is a major concern that should be considered for mass concrete structures from the standpoints of structures and materials. Thus, for mass concrete structures in which thermal-stress-induced cracks are prone to occur, considerations should include not only rational structure types and reasonable construction sequences but also suitable cement materials. Supersulfated cement (SSC) is particularly suitable for use in the internal part of hydraulic structure mass concrete due to its slow hydration rate and very low hydration heat.

For the cement industry, re-use binders are used for waste materials from other industries; these binders are effective for energy-saving emissions reduction to reduce energy consumption and CO_2_ emissions [1,2]. SSC is a type of cement with little or no clinker, which contains nearly 80% ground granulated blast furnace slag, 15% gypsum as the sulfate activator and less than 5% clinker or lime as the alkali activator [3]. The hydration heat of SSC is much lower than that of ordinary Portland cement; therefore, SSC is thought to have promising potential for industrial applications [4].

For mass concrete structures, reasonable joint arrangement and stratification are significant. Concrete is poured in layers, which offers convenience in construction and improves the heat dissipation conditions. Moreover, pre-cooling techniques, such as cooling aggregates and reducing cement content, and the post-cooling of embedded cooling pipes are widely used. The first successful post-cooling technique was employed during the construction of the Hoover Dam [5] and is still used today. Considering the advantage of low hydration heat of SSC concrete, water-cooling is likely to be cancelled, which can bring about construction cost-savings for mass concrete structures.

In most cases, proper materials may be selected to satisfy the strength requirements; reasonable construction and cooling schedules may also be established according to regular calculations. However, because regular calculations typically simplify the hydration process and difficulties arise in predicting concrete properties such as the modulus of elasticity [6], cracks may still occur unpredictably in mass concrete structures and affect their durability and service performance. Therefore, it is necessary to simulate the thermal field in a proper manner that considers the inner temperature, hydration heat generation rate and strength development rate in mass concrete so the effective temperature control measures can be adopted to prevent cracking.

Since the effect of the temperature and the cement hydration can promote or hinder in the concrete formation of micro-cracks which in turn influence the behavior of the material itself, the understanding of this phenomenon is very relevant. Indeed, recent works [7,8] have shown how the presence of micro-cracks can result, sometimes, in the improvement of the dissipative capacity of mass concrete structures. Thus, the accurate prediction of thermal-mechanical behaviors is of great significance for concrete structures.

In general, computer simulations (typically based on the finite element method) allow us to precisely evaluate the thermal evolution of early-age concrete by considering the hydration degree as an internal variable and other related parameters in describing the thermo-chemical-mechanical coupling process [9,10,11,12,13,14,15]. Cervera *et al.* proposed a coupled thermo-chemo-mechanical model for the behavior of early-age concrete that can be implemented into the finite element method and predict the evolution of the hydration degree and heat over time [16,17,18]. Lackner *et al.* found a significant linear correlation between hydration degree and the intrinsic material functions for the fracture energy [19]. Gawin *et al.* proposed a model that not only took the thermal and chemical fields but also the water diffusion process coupled effects into consideration [20]. Taking various chemical reactions explicitly into account, Di Luzio and Cusatis introduced a new hygro-thermo-chemical model for high-performance concrete suitable for the analysis of moisture transport and heat transfer [21,22].

In this paper, one type of environment-friendly cement, SSC, is introduced, and its hydration properties are studied based on hydration heat liberation rate data. A thermo-chemical-mechanical coupled hydration model was adopted to simulate the temperature and stress field in a relatively precise way for mass concrete structures. A multi-scale framework is employed to obtain the nonlinear thermal and mechanical characteristics. The simulation model is applied in a case study on a monolith of an arch dam.

## 2. Thermo-Chemical-Mechanical Coupled Model

### 2.1. Conventional Method for Concrete Hydration Heat and Elastic Modulus Estimation

For mass concrete structures, the hydration heat is the main heat source, which is significant in simulating the temperature and stress field. The conventional method to calculate the hydration heat of concrete is to employ the adiabatic temperature rise model, which considers the hydration heat as a function (usually composite exponential or hyperbolic type) of hydration time. For mass concrete structures, one of the most widely used models is given by [23]:

(1) $$Q(t)=Q_{\infty }(1-e^{-at^{b}}),$$

where Q(t) is the hydration heat at time *t,* and $Q_{\infty }$ is the ultimate hydration heat; *a* and *b* are material parameters that can be calibrated with adiabatic test data.

Adiabatic tests are conducted with an initial temperature of 20–25 °C. From the viewpoint of cement hydration kinetics, concrete hydration occurs quickly under adiabatic conditions. Thus, variations in the adiabatic temperature rise are generally too low to measure after 28 days [24]. Therefore, using the above model, hydration heat evolution presents a fat trend after of 28 days, which shows a different method than standard engineering practices. Owing to lower placing temperatures (approximately 10 °C or lower) in mass concrete structures, hydration processes are retarded, then hydration heat is still generated at a considerably slower rate after 28 days.

Similar to the hydration heat calculation, the elastic modulus of mass concrete is described as follows:

(2) $$E(t)=E_{\infty }(1-e^{-\alpha t^{\beta }}),$$

where E(t) is the elastic modulus at time *t,* and $E_{\infty }$ is the ultimate elastic modulus; *α* and *β* are material parameters.

### 2.2. Long-Term Hydration Model

Hydration models based on the Arrhenius concept are widely accepted by researchers because they use rigorous thermodynamic theory and are consistent with experimental data [25]. According to Arrhenius’s law, the time-dependent hydration progress could be described as in Equation (1). In the modeling, the hydration rate is related to the temperature and the chemical affinity $A_{\xi }(\xi )$:

(3) $$\frac{\partial \xi }{\partial t}=A_{\xi }(\xi )\exp(-\frac{E_{a}}{RT}),$$

where $E_{a}$ is the activation energy of the reaction, and *R* is a constant for ideal gases. Cervera *et al.* developed an analytical form of the normalized affinity based on thermodynamics [17]. The free energy for the thermo-chemical system can be divided into three parts: thermal contribution, thermo-chemical coupling, and chemical contribution. The chemical contribution in this work will be considered as a quartic function instead of a cubic function. Similar to the strategy presented in [17], a fixed form of the chemical affinity can be derived as follows [26]:

(4) $$A_{\xi }(\xi )=\beta_{1}(\beta_{2}+\beta_{3}\xi +\xi^{2})(\xi_{\infty }-\xi )\exp(-\bar{\eta }\frac{\xi }{\xi_{\infty }}),$$

where $\beta_{1}$, $\beta_{2}$ and $\beta_{3}$ are material coefficients, $\xi_{\infty }$ is the ultimate hydration degree, and $\bar{\eta }$ represents the viscosity due to micro-diffusion of the free water through the already-formed hydrates.

The advantage of the fixed hydration model is discussed in [26]. Notably, $\xi_{\infty }$ is set as one in this paper because the construction periods of mass concrete structures are quite long (typically several months or even years). In this way, the number of arguments the function takes can be reduced. Parameters $\beta_{1}$, $\beta_{2}$, $\beta_{3}$ and $\bar{\eta }$ can be calibrated using the experimental results.

### 2.3. Transient Heat Transfer Process

The transient heat transfer process of concrete can be described as follows:

(5) $$\rho C\frac{\partial T(x,y,z,t)}{\partial t}=\lambda_{T}\Delta T(x,y,z,t)+\frac{\partial Q}{\partial t},$$

(6) $$\frac{\partial Q}{\partial t}{=Q}_{\infty }\frac{\partial \xi }{\partial t},$$

where ρ, C, $\lambda_{T}$, Δ and $Q_{\infty }$ are the density, volumetric heat capacity, thermal conductivity coefficient, Laplacian operator and the final volumetric heat of hydration, respectively. The latent heat release due to concrete hydration is actually a nonlinear and thermally dependent process.

### 2.4. Thermal-Creep Stress Calculation

According to the studies of Di Luzio and Cusatis [27], based on the assumption that the strain additivity holds, the composition of concrete strain mainly includes elastic strain, cracking strain, creep (or viscoelastic) strain, thermal strain, and shrinkage strain [28]:

(7) $$\dot{\varepsilon }(t)={\dot{\varepsilon }}_{e}+{\dot{\varepsilon }}_{cr}+{\dot{\varepsilon }}_{c}+{\dot{\varepsilon }}_{T}+{\dot{\varepsilon }}_{sh},$$

where ${\dot{\varepsilon }}_{e}$, ${\dot{\varepsilon }}_{c}$, ${\dot{\varepsilon }}_{cr}$, ${\dot{\varepsilon }}_{th}$ and ${\dot{\varepsilon }}_{sh}$ are the elastic strain rate, cracking strain rate, creep strain rate, thermal strain rate and shrinkage strain rate, respectively.

Based on engineering facts, the thermal stress of mass concrete structures is usually calculated by a viscoelastic model. This paper aims to conduct contrastive analysis of conventional computations with the coupled hydration model; thus, thermal-chemical coupled effects which used to be ignored in engineering practice are our major considerations. The constitutive law in this paper for the mechanical behavior is the conventional one for engineering calculations, which is simplified.

Accordingly, the stress evolution can be described as follows:

(8) $$\dot{\sigma }=E_{\left(\xi \right)}{\dot{\varepsilon }}_{e}=E_{\left(\xi \right)}(\dot{\varepsilon }-{\dot{\varepsilon }}_{c}-{\dot{\varepsilon }}_{T}-{\dot{\varepsilon }}_{sh}).$$

#### 2.4.1. Shrinkage

Total shrinkage of concrete may be composed of three parts: the autogeneous shrinkage, carbonation shrinkage and drying shrinkage. In good-quality concrete, carbon dioxide penetrates only a very thin surface layer, and the carbonation shrinkage is usually small and can be neglected [29].

As concrete hardens, the pore structure would change, which leads to the development of autogenous shrinkage. As a consequence, autogenous shrinkage is closely related to the evolution of hydration in the material. Experimental results indicate that autogenous shrinkage evolution is in a linear relationship with the hydration degree [10], so autogenous shrinkage $\varepsilon_{as}$ can be modeled by linear function or piecewise linear function [30]:

(9) $${\dot{\varepsilon }}_{as}={-(k}_{a}\dot{\xi }+k_{b})1(\xi >\xi_{0}),$$

where *k_a_* and *k_b_* are material parameters, **1** is the unit tensor and $\xi_{0}$ is a threshold.

Considering that the drying shrinkage behaviors usually take place on the surface of structures, and humidity diffusion coefficient is quite small (under the environment with relative humidity of 50%, it may take a month to reach a drying depth of 7 cm, which is far smaller than the size of mass concrete structures); as a consequence, for the inner part of mass concrete structures, the humidity can be treated as constant [23]; thus, the drying creep effect is usually neglected for mass concrete structures.

#### 2.4.2. Creep

The mass concrete creep can be modeled by means of the solidification theory. Bofang Zhu compared the generalized Kelvin rheological model with the Dirichlet series of creep compliance and summarized a creep model with eight parameters, which has been widely adopted in China [23]. The formulations can be deduced from Kelvin chain model (with two units in series):

(10) $$\left\{ \begin{array}{l} J(t,\tau )=\frac{1}{E(\tau )}+C(t,\tau )\\ C(t,\tau )=\sum_{i=1}^{2}\left(A_{i}+B_{i}\tau^{C_{i}})[1-e^{-D_{i}(t-\tau )}\right] \end{array}\right.,$$

where *t* is the time, τ is the age of concrete, E(τ) is the elastic modulus of the concrete, J(t,τ) is the creep compliance function, C(t,τ) is the creep function, and $A_{i}–D_{i}$ are material parameters.

Since the creep experiments usually show the total creep strain in practice, and as has been discussed in Section 2.4.1, the humidity is usually treated as constant within mass concrete structures. In this work, additional creep due to drying (drying creep), also called the stress-induced shrinkage (or Pickett effect) [31], would be simplified, as well.

#### 2.4.3. Thermal Strain

The thermal strain $\varepsilon_{th}$ is related to the temperature variation:

(11) $${\dot{\varepsilon }}_{th}=\alpha_{t}\dot{T}1,$$

where $\alpha_{t}$ is the thermal dilatation coefficient (kept constant), and **1** is the unit tensor.

### 2.5. Thermal and Mechanical Characteristics

In a fully coupled thermo-chemical-mechanical model, the hydration degree can be used as an intermediate variable to determine the thermal or mechanical characteristics. In this work, the heat capacity and thermal conductivity of concrete are estimated using a homogenization scheme that is well-suited for heterogeneous material.

#### 2.5.1. Heat Capacity

Given the heat capacity and volume fraction values of cement, water and aggregates, the heat capacity of the matrix can be estimated using the law of mixtures [32]:

(12) $$\left\{ \begin{array}{l} c_{freshpaste}=c_{water}f_{water}+c_{cement}f_{cement}\\ c_{paste}(\xi )=c_{freshpaste}\left\{1-A\left[1-\exp(-B\xi )\right]\right\}\\ c_{concrete}=c_{paste}f_{paste}+c_{agg}f_{agg}+c_{filler}f_{filler} \end{array}\right.,$$

where $c_{freshpaste}$, $c_{paste}(\xi )$, $c_{water}$, $c_{cement}$, $c_{filler}$, and $c_{agg}$ are the heat capacity (per unit volume) of fresh paste, paste in the hydration process, water, cement, the filler, and the aggregates, respectively. $f_{water}$, $f_{cement}$, $f_{paste}$, $f_{filler}$, and $f_{agg}$ are the volume fraction of water, cement, the paste, the filler and the aggregates, respectively. ***A*** and ***B*** are material constants; in Bentz’s study [32], these values were measured with *w/c* varying between 0.3 and 0.5.

#### 2.5.2. Thermal Conductivity

The paste matrix is made of water and cementitious powder, so a reasonable approximation of thermal conductivity can be calculated from the well-known Hashin–Shtrikman (H-S) bounds. An estimate can be determined from the measured thermal conductivities ($\lambda_{1}$,$\lambda_{2}$) and phase volume fractions ($f_{1}$,$f_{2}$). For $\lambda_{2}\geq \lambda_{1}$, the Hashin–Shtrikman lower ($\lambda_{l}$) and upper ($\lambda_{u}$) bounds for the thermal conductivity of a two-phase composite are given by [32]:

(13) $$\left\{ \begin{array}{l} \lambda_{l}=\lambda_{1}+\frac{f_{2}}{\frac{1}{\left(\lambda_{2}-\lambda_{1}\right)}+\frac{f_{1}}{3\lambda_{1}}}\\ \lambda_{u}=\lambda_{2}+\frac{f_{1}}{\frac{1}{\left(\lambda_{1}-\lambda_{2}\right)}+\frac{f_{2}}{3\lambda_{2}}} \end{array}\right.,$$

An estimate can then be determined by the average of the H-S bounds:

(14) $$\lambda_{hom}=\frac{\lambda_{l}+\lambda_{u}}{2},$$

where $\lambda_{hom}$ is the effective thermal conductivity of the composite.

According to [32,33], the thermal conductivity of cement paste is a function of volume fraction rather than hydration degree. Thus, this work ignores the nonlinear behaviors of thermal conductivity.

#### 2.5.3. Aging Degree

Experimental evidences show that the evolution of the concrete strength depends not only on the degree of hydration, but also on the kinetics of the hydration reaction. On the basis of this evidence, Cervera *et al.* proposed an aging degree model to link strength evolution with hydration degree and current temperature, and the aging degreeκ, can be defined as [17,34]:

(15) $$\left\{ \begin{array}{l} \dot{\kappa }={\left(\frac{T_{\max}-T}{T_{\max}-T_{ref}}\right)}^{n_{\kappa }}(B_{\kappa }-2A_{\kappa }\xi )\dot{\xi }\begin{array}{cc} & \left(\xi \geq \xi_{set}\right) \end{array}\\ \dot{\kappa }=0\begin{array}{cccc} & & & \left(\xi <\xi_{set}\right) \end{array} \end{array}\right.,$$

where *T_max_* represents the maximum temperature during hydration of concrete which is possibly under standard conditions (100 °C); *T_ref_* is the reference temperature for the experimental calibration of the aging model; $\xi_{set}$ defines the value of the hydration degree at the end of the setting phase; and $n_{\kappa }$, $B_{\kappa }$ and $A_{\kappa }$ are material parameters.

#### 2.5.4. Compressive and Tensile Strength

The Young’s modulus values vary due to the aging degree’s evolution as follows:

(16) $$\left\{ \begin{array}{l} E_{\left(\xi \right)}^{c}=\kappa (T,\xi )E_{\infty }^{c}\\ E_{\left(\xi \right)}^{t}=\kappa {\left(T,\xi \right)}^{2/3}E_{\infty }^{t} \end{array}\right.,$$

where $E_{\left(\xi \right)}^{c}$ and $E_{\left(\xi \right)}^{t}$ are compressive and tensile strength at the degrees of hydration ξ and $\xi_{\infty }$, respectively, $E_{\infty }^{c}$ and $E_{\infty }^{t}$ are compressive and tensile strength, respectively [17,35].

#### 2.5.5. Young’s Modulus

The Young’s modulus varies due to the aging degree’s evolution as follows:

(17) $$E_{\left(\xi \right)}=\kappa {\left(T,\xi \right)}^{1/2}E_{\infty },$$

where $E_{\left(\xi \right)}$ and $E_{\infty }$ are Young’s modulus at degree of hydration ξ and $\xi_{\infty }$, respectively.

#### 2.5.6. Poisson’s Ratio

Based on the work of De Schutter *et al.* [36,37], the Poisson’s ratio of concrete can be estimated as follows:

(18) $$\mu_{\left(\xi \right)}=\left[\mu_{\infty }-0.49\exp(-10)\right]\sin(\frac{\pi }{2}\xi )+0.49\exp(-10\xi ),$$

where $\mu_{\left(\xi \right)}$ and $\mu_{\infty }$ are Poisson’s ratio at the degrees of hydration ξ and $\xi_{\infty }$, respectively.

## 3. Validation

### 3.1. Calibration Method of the Hydration Model

As discussed in Section 2, the value of thermo-chemical-mechanical characteristics are associated with the temperature or hydration degree values. In this section, based on the experimental values, the hydration model in this paper is numerically verified, and the parameters are calibrated.

At present, the kinetic parameters of the hydration reaction of concrete were achieved by testing temperature or the rate of hydration heat evolution with the adiabatic tests with the following equations:

(19) $$\frac{1}{Q}=\frac{1}{Q_{\infty }}+\frac{t_{0.5}}{Q_{\infty }(t-t_{0})},$$

(20) $$\xi (t)=\frac{Q(t)}{Q_{\infty }},$$

where Q is the hydration heat evolution quantity; $Q_{\infty }$ is the ultimate hydration heat evolution quantity; ξ(t) is the hydration degree; $t_{0}$ is the end time of the induction period, $t_{0.5}$ is the time moment when the hydration degree is 0.5. Once the hydration heat evolution is obtained, Equations (18) and (19) are combined, and an inverse analysis is adopted to determine the parameters of the hydration model.

### 3.2. Fly Ash Concrete

**Figure 1:**
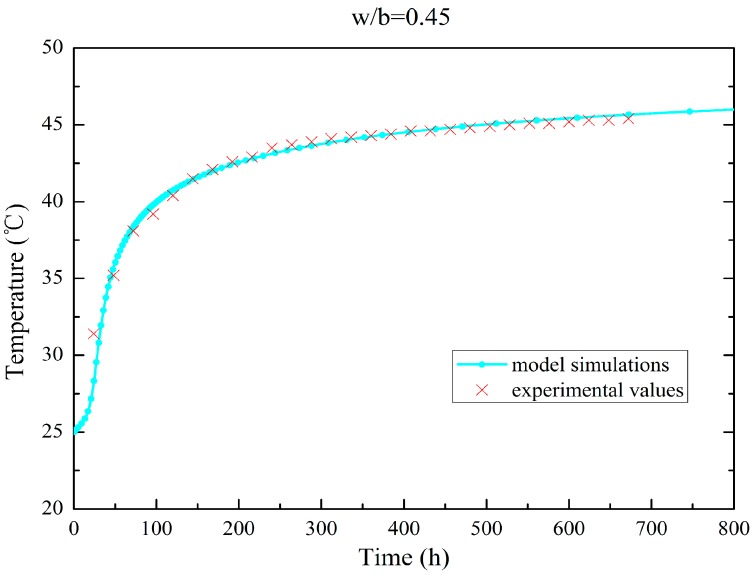
Temperature evolution for adiabatic test of fly ash concrete (w/b = 0.45).

**Figure 2:**
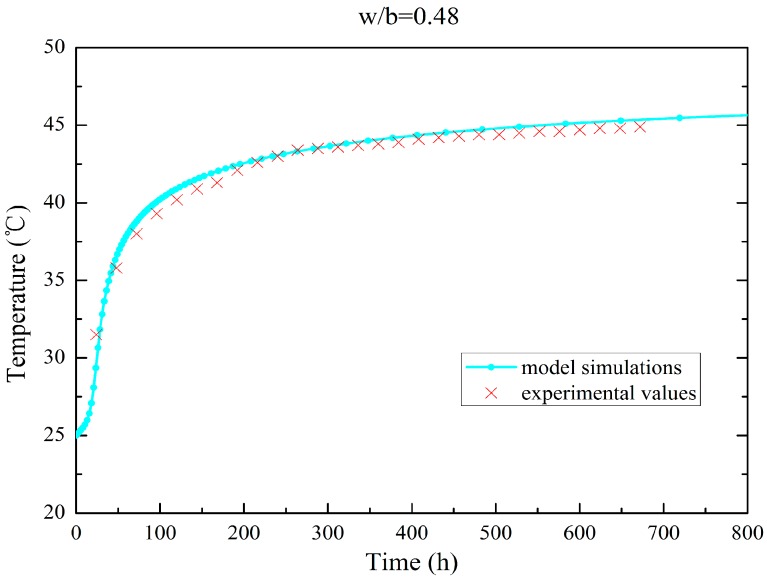
Temperature evolution for adiabatic test of fly ash concrete (w/b = 0.48).

Using the approach in Section 3.1, the temperature evolution of the simulations and adiabatic tests on concrete samples are plotted in Figure 1 and Figure 2. The results show that the hydration model simulation conforms to the experimental values (the error is no more than 2%). Table 1 and Table 2 summarize the mass fractions of the components and the thermal parameters of two types of fly ash concrete with degree four of gradation. Table 3 lists the results of the parameter calibration for the hydro-thermal model.

**Figure 3:**
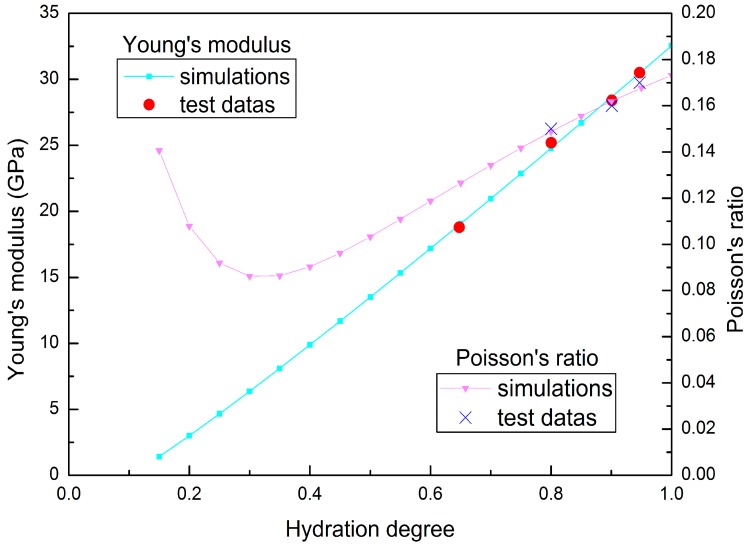
Young’s modulus and Poisson’s ratio evolution for isothermal test of fly ash concrete (w/b = 0.45).

**Figure 4:**
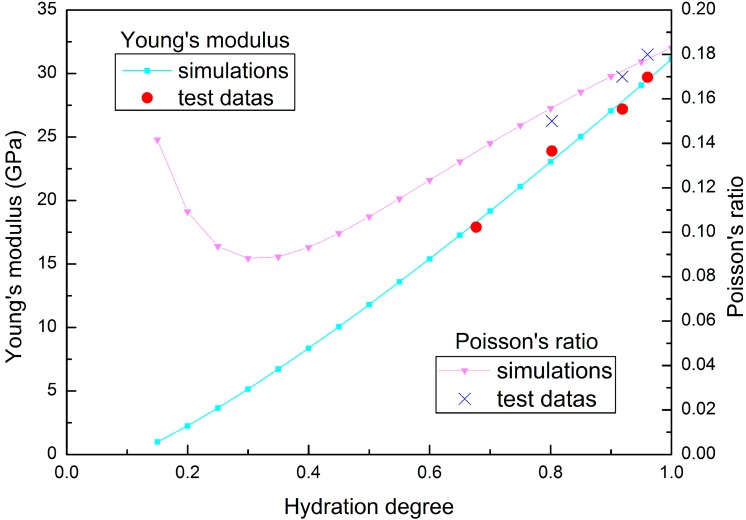
Young’s modulus and Poisson’s ratio evolution for isothermal test of fly ash concrete (w/b = 0.48).

Mechanical tests on concrete are typically conducted under isothermal conditions; therefore, the hydration process is simulated under isothermal condition in this research, so the mechanical characteristics can be linked to the hydration degree rather than to time. Young’s modulus and the Poisson’s ratio evolution for isothermal test of fly ash concrete specimen are plotted in Figure 3 and Figure 4.

**Figure 5:**
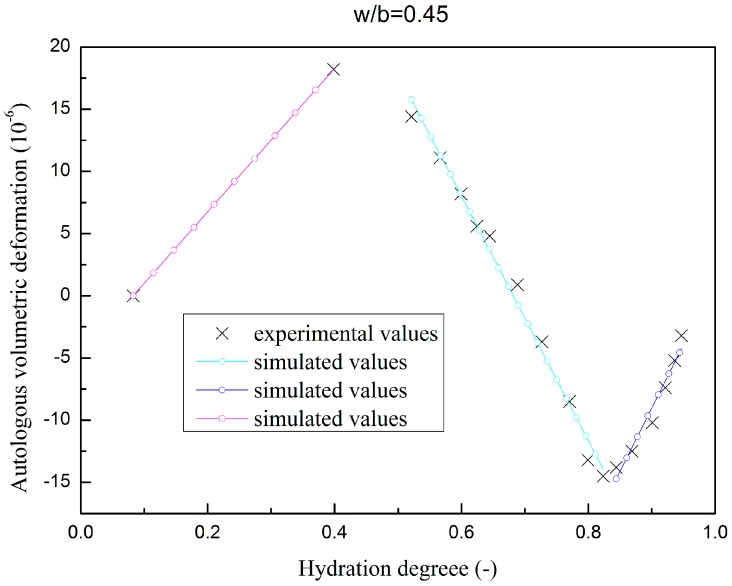
Autologous volumetric deformation evolution of fly ash concrete (w/b = 0.45).

**Figure 6:**
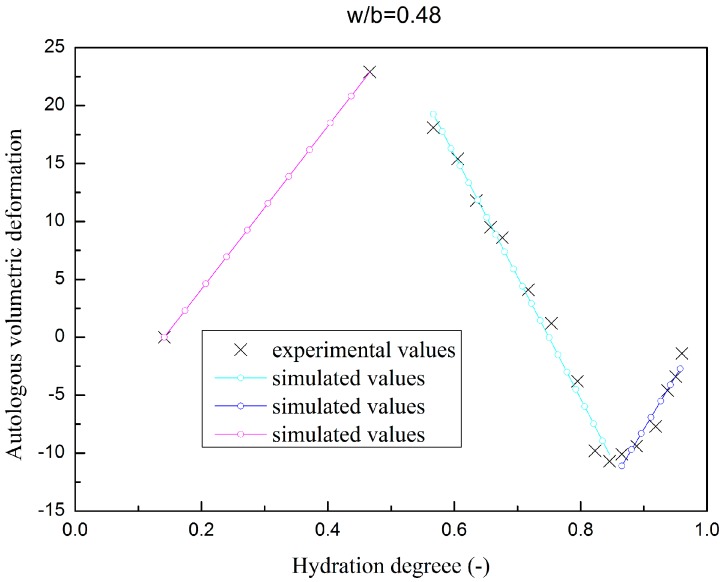
Autologous volumetric deformation evolution of fly ash concrete (w/b = 0.48).

The autologous volumetric deformation of fly ash concrete specimen are plotted in Figure 5 and Figure 6, as has discussed in Section 2.4.1; piecewise linear functions are adopted to simulate the autologous volumetric deformation.

### 3.3. SSC Concrete

The hydration properties of SSC are different from slag Portland cement and ordinary Portland cement [38]. SSC achieves a lower heat release compared to slag Portland cement and ordinary Portland cement. Conversely, slag cannot be simulated without a suitable alkaline solution, so the second exothermal peaks of the SSC present later than that of slag Portland cement and ordinary Portland cement. On the basis of the experimental studies in [3,38], the hydration properties are simulated in a numerical method.

**Figure 7:**
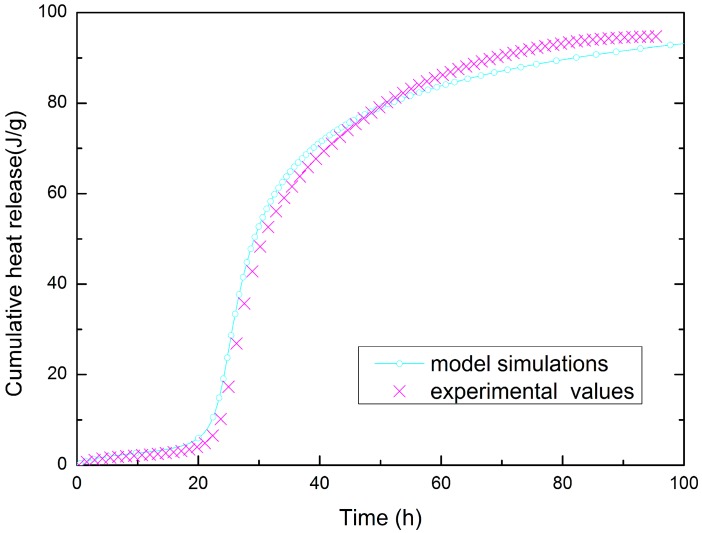
Hydration heat quantity curves for adiabatic test of SSC (w/c = 0.40).

Figure 7 shows the cumulative heat release quantity of the model simulations and experimental values for SSC; the simulations are in accordance with the experimental data (the error is within 4%). The parameters of the hydrothermal model of SSC are listed in Table 4.

The hydration properties can be analyzed, given the kinetic parameters of the hydration reaction of the cement paste and the composition of concrete, which is listed in Table 5. Assuming the aggregates have no impact on the hydration process, the ultimate hydration heat evolution quantity can be obtained as below:

(21) $$Q_{\infty }=Q_{\infty }^{e}f_{cement},$$

where $Q_{\infty }^{e}$ is the ultimate hydration heat evolution quantity of the cement paste, and $f_{cement}$ is the fraction of cement paste in the concrete.

**Figure 8:**
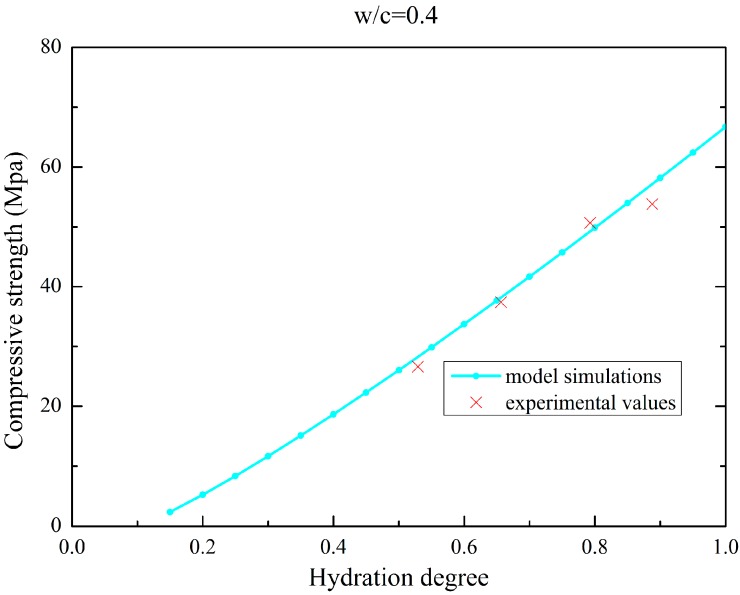
Compressive strength evolution for isothermal test of SSC (w/c = 0.40).

The compressive strength evolution for isothermal tests of the SSC specimen is plotted in Figure 8.

## 4. Engineering Application

### 4.1. Project Background and Assumptions

The Dagangshan Hydropower Station is one of the largest hydropower projects built on the main stream of Dadu River in Southwest China. The water retaining structure of the Dagangshan water complex is a double-curvature arch dam with a maximum height of 210 m, 29 dam sections, and total concrete dosages of approximate 3,220,000 m^3^ [39]. In this study, one of the dam sections, #18, was selected for the thermo-chemical-mechanical analysis.

For mass concrete structures, thermal stresses are a major concern, and temperature control measures are Gordian techniques in the construction period. There are multiple factors that impact thermal stress, such as concrete material properties, boundary conditions, construction schedule and temperature control measures.

The cooling effects of the cooling pipes can be estimated according to [24,40,41]; however, in this work for simplification, the cooling effects are not included in the calculations.

### 4.2. FE Model Description and Initial Conditions

**Figure 9:**
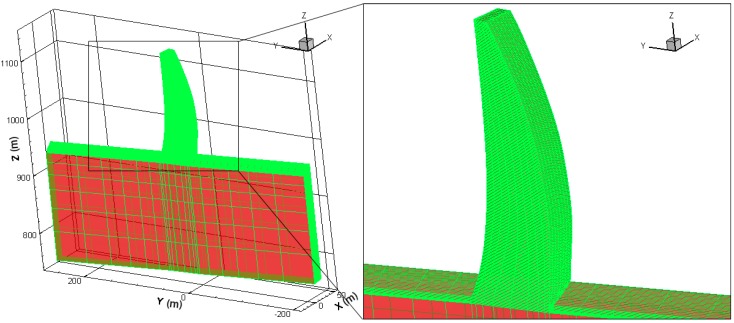
3D FE mesh model of the #18 dam section of Dagangshan Dam.

The #18 dam monolith located on the riverbed was selected as a typical structure to conduct the thermo-chemical-mechanical analysis. The 3D FE mesh model for the simulation is presented in Figure 9. A birth and death technique is adopted to address the existence and nonexistence of the concrete. Corresponding elements in the finite element model are made active when a fresh concrete slab was poured. Thus, the incremental pouring process of concrete can be simulated in the FE model.

The construction monolith #18 of the Dagangshan Dam began on 11 March 2012, and it was completed on 25 September 2014. Next, 72 layers of concrete gradually rose with the progress of construction. The initial temperature of the concrete was assumed the placing temperature [23], according to the monitoring records.

### 4.3. Boundary Conditions

In normal conditions, the construction of the selected monolith was always behind the adjacent monoliths; the boundary condition for both sides was assumed adiabatic. The upstream and downstream surfaces of the placed concrete as well as the top surface of the concrete block and foundation were assumed directly exposed to the outside environment, where heat convection and radiation occurred.

Now that the project has been completed, the distribution characters of the air temperature can be simulated based on the measured data.

**Figure 10:**
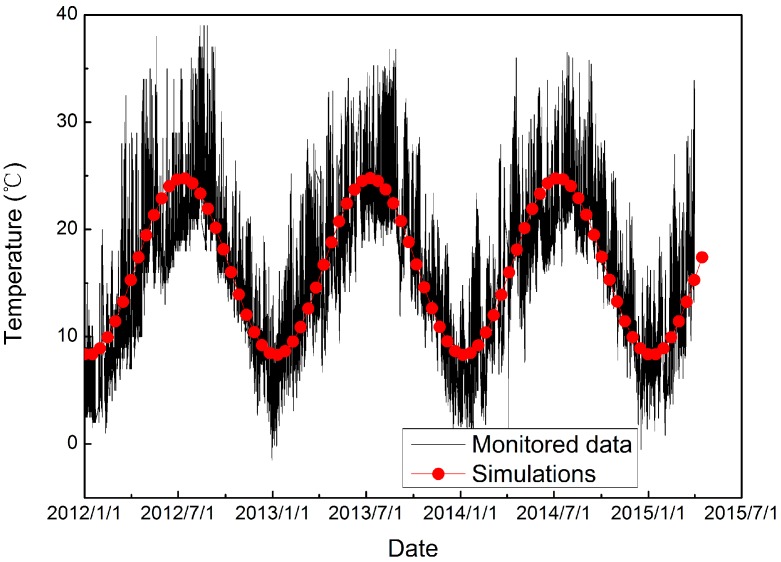
Monitored and simulated air temperature values.

According to the monitoring temperature of the meteorological station at the Dagangshan Dam site, the outside air temperature (*T_a_*) of the measured temperature values (Figure 10) can be illustrated by the following fitted function:

(22) $$T_{a}=16.542+8.25\cos\left[\frac{2\pi }{12}\left(\frac{12t}{365}-6.25\right)\right].$$

### 4.4. Results and Discussion

**Figure 11:**
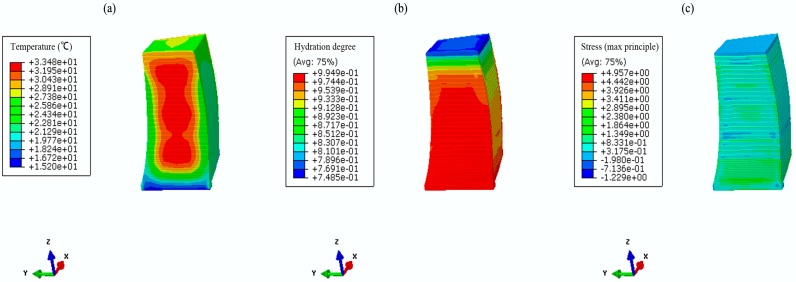
The coupled thermo-chemical-mechanical fields computations on 5 June 2013. (**a**) temperature (°C); (**b**) hydration degree (-); (**c**) first principle stress (MPa).

**Figure 12:**
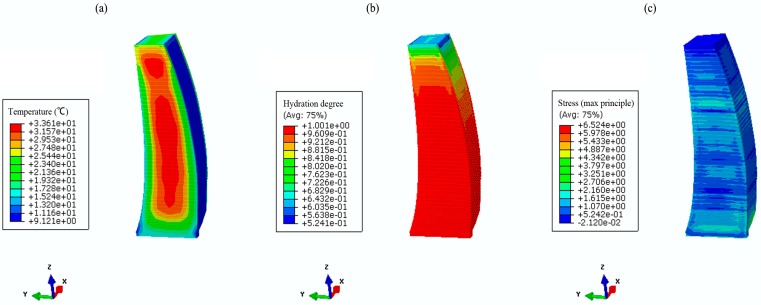
The coupled thermo-chemical-mechanical fields computations on 13 March 2014. (**a**) temperature (°C); (**b**) hydration degree (-); (**c**) first principle stress (MPa).

**Figure 13:**
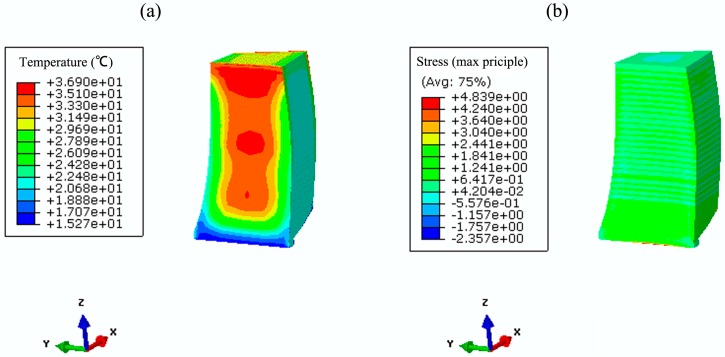
Conventional computations of the temperature and stress field on 5 June 2013. (**a**) temperature (°C); (**b**) first principle stress (MPa).

**Figure 14:**
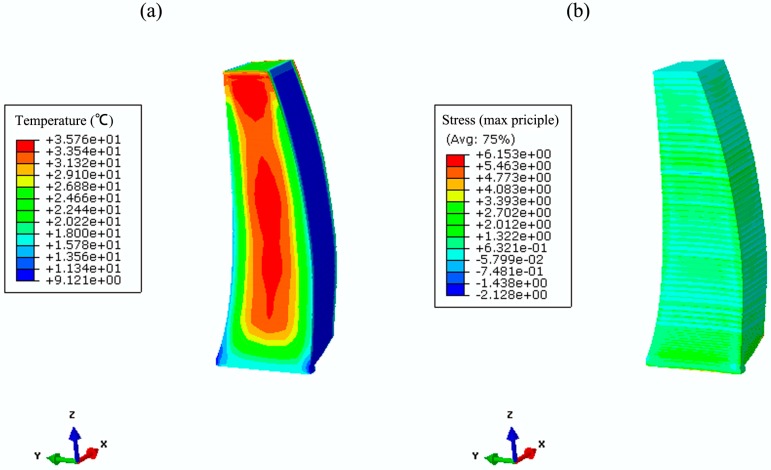
Conventional computations of the temperature and stress field on 13 March 2014. (**a**) temperature (°C); (**b**) first principle stress (MPa).

Based on the parameter studies in Section 2 and numerical validation in Section 3, temperature and stress fields are simulated using both the conventional method and the hydration model. Figure 11 and Figure 12 show the coupled thermo-chemical-mechanical fields simulations on different dates, accordingly; Figure 13 and Figure 14 show the solutions of temperature and stress field using the conventional model. The results indicate that temperature distribution only differed within the relatively fresh concrete layers; temperature computations of the coupled model are smaller than that of the conventional model. However, different distribution patterns are present in the stress fields. Moreover, the maximum temperature resulting from the coupled model is lower than that of the conventional model, mainly because the hydration heat release process in the coupled model takes a long period, which corresponds to the engineering facts.

**Figure 15:**
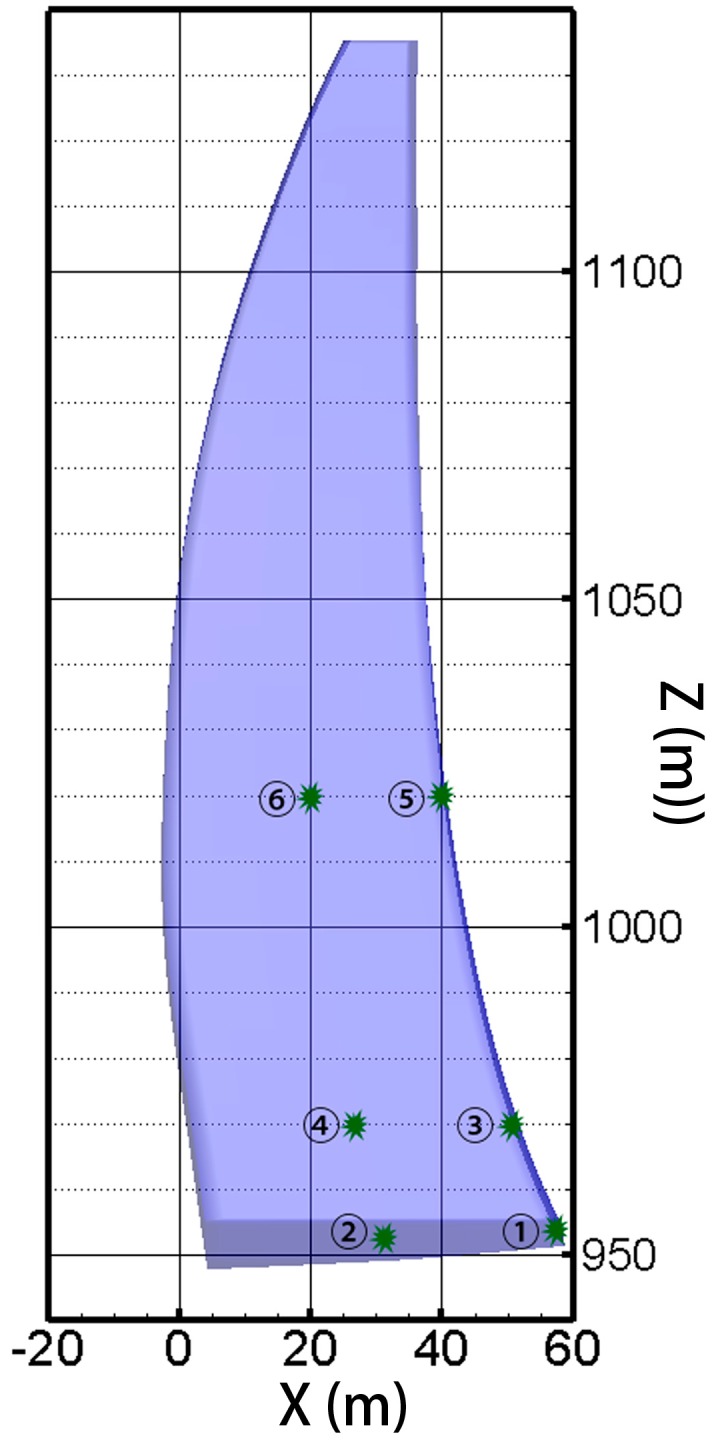
The position distribution of typical points.

**Figure 16:**
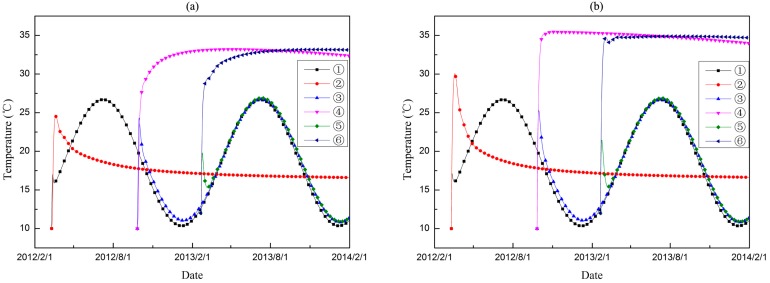
The temperature histories of different typical points. (**a**) thermo-chemical-mechanical coupled model; (**b**) conventional model.

**Figure 17:**
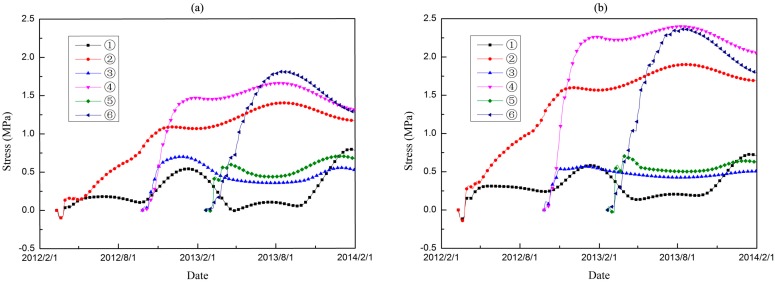
The max principle stress histories of different typical points. (**a**) thermo-chemical-mechanical coupled model; (**b**) conventional model.

The temperature and stress evolution processes are discussed for further comparative analysis. Six typical point are selected, and points ①, ③ and ⑤ are on the downstream profile of the dam section, whereas, points ②, ④ and ⑥ are distributed over the midplane. Points ① and②, points ③ and ④, and points ⑤ and ⑥ are on the three different elevations. The position distribution of the typical points is shown in Figure 15. Thus, the pouring layers of the typical points are different, as is the age of the concrete. Figure 16 shows the temperature history of each typical point, and Figure 17 shows the maximum principle stress histories of the typical points.

For the nodes on the downstream profile of the dam section, the temperature rises to a certain extent and then changes with air temperature. Compared with the conventional model, the temperature prediction values of the coupled model present a slower growth trend inside the mass concrete. The temperature procedure differences give rise to differentiate the effective stress development history. For points on the surface, the calculated stress values of the coupled model are relatively larger than that of conventional model. However, the opposite occurs for the inner part of dam section.

**Figure 18:**
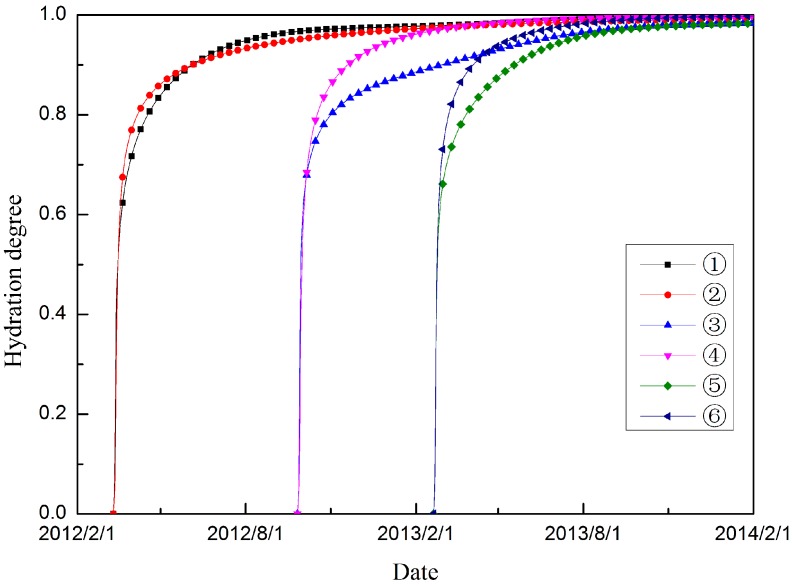
Hydration degree histories of different typical points.

Figure 18 shows the hydration degree histories of the selected typical points. Owing to the existence of temperature heterogeneity, the hydration degree is present in an inhomogeneous distribution within the mass concrete structure. The poor heat emission condition of the inner part of dam accelerates the hydration process of the concrete in these regions. Thus, the growth of cement strength of concrete on the upstream and downstream surfaces lags behind that of the inner regions.

**Figure 19:**
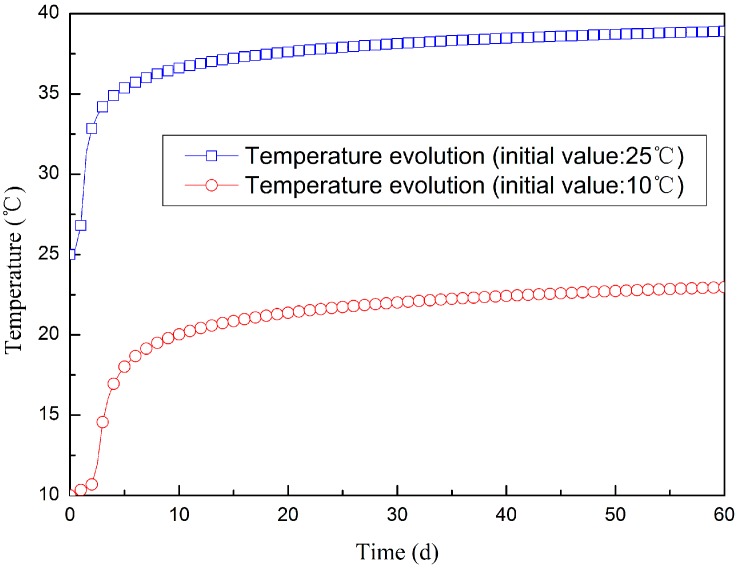
Temperature evolution of SSC concrete under adiabatic conditions.

Figure 19 shows the temperature evolution of the SSC concrete under adiabatic conditions. The results show that the adiabatic temperature of the SSC concrete increase less than 15 °C. For mass concrete structures such as the Dagangshan Dam, though pre-cooling techniques are adopted, water cooling techniques are still required to control the maximum temperature. For example, for Dagangshan Dam, the design maximum temperatures are 27 °C (for the foundation constrained region) and 30 °C (for the non-constraint region). Therefore, when using SSC concrete, the maximum temperature of mass concrete can satisfy the design requirements without the need of water cooling.

## 5. Conclusions

In view of the disadvantages of the conventional temperature prediction model, this work introduces a fixed hydration model to study the non-linear thermo-chemical-mechanical responses in mass concrete structures. Focusing on the chemical-physical responses of mass concrete structures, the authors conducted numerical simulations and parameter studies. The major achievements are as follows:

- The conventional heat release model of mass concrete only relates the heat liberation rate to time, which ignores the effect of real-time temperature on the hydration process. To overcome the problem, a chemical-physical coupled model is introduced.
- Based on the chemical-physical coupled model, the hydration properties of fly ash concrete and SSC concrete are studied.
- A multi-scale framework is employed to obtain the nonlinear thermal characteristics. Using a homogenization scheme that is well suited for heterogeneous materials, the hydration process of mass concrete structures can be estimated.
- Contrastive analysis of the coupled model and the conventional model are conducted, based on an engineering project.
- Temperature prediction values of the conventional model and the coupled hydration model show a difference, especially at an early age when cracking is prone to occur within mass concrete structures.
- The cooling effect of the cooling pipes used inside the mass concrete was neglected. Thus, the chemical-physical responses under actual working conditions should be scrutinized in future research.
- When using proper materials such as super sulfated slag concrete, water cooling is likely to be abrogated, which can induce cost-savings.

## Figures and Tables

**Table 1:** Component mass of fly ash concrete samples with varying water-binder ratios.

w/b	Composition of Concrete (kg/m^3^)								Set-Retarding Superplasticizer	Air Entraining Agent
	Water	Cement	Fly Ash	Sand	Stone Small	Stone Middle	Stone Large	Stone Extra		
0.45	85	123	66	558	318	318	477	477	0.7%	0.02%
0.48	85	115	62	572	317	317	476	476	0.7%	0.02%

**Table 2:** Thermal parameters of the components.

$\rho_{filler}$ (kg·m^−3^)	$\rho_{water}$ (kg·m^−3^)	$\rho_{cement}$ (kg·m^−3^)	$\rho_{fly}$ (kg·m^−3^)	$\rho_{agg}$ (kg·m^−3^)
1040	1000	3240	2380	2620
$c_{filler}$ **(kJ·m^−3^·K^−1^)**	$c_{water}$ **(kJ·m^−3^·K^−1^)**	$c_{cement}$ **(kJ·m^−3^·K^−1^)**	$c_{fly}$ **(kJ·m^−3^·K^−1^)**	$c_{agg}$ **(kJ·m^−3^·K^−1^)**
980	4180	2415	2190	1886
$\lambda_{filler}$ **(J·h^−1^·m^−1^·K^−1^)**	$\lambda_{water}$ **(J·h^−1^·m^−1^·K^−1^)**	$\lambda_{cement}$ **(J·h^−1^·m^−1^·K^−1^)**	$\lambda_{fly}$ **(J·h^−1^·m^−1^·K^−1^)**	$\lambda_{agg}$ **(J·h^−1^·m^−1^·K^−1^)**
2160	2174	5580	840	8800

**Table 3:** Parameters for the hydro-thermal model of fly ash concrete.

Parameters	$\beta_{1}$ (10^7^ h^−1^)	$\beta_{2}$ (10^−4^)	$\beta_{3}$ (-)	$\xi_{\infty }$ (-)	$\bar{\eta }$ (-)	$Q_{\infty }$ (J·kg^−1^)	$E_{a}/R$ (K)
w/b = 0.45	6.4	9.2	−0.016	1	9.6	19,200	5000
w/b = 0.48	7.5	8	−0.012	1	9.5	18,550	5000

**Table 4:** Parameters of the hydrothermal model of SSC paste.

Parameters	$\beta_{1}$ (10^8^ h^−1^)	$\beta_{2}$ (10^−4^)	$\beta_{3}$ (-)	$\xi_{\infty }$ (-)	$\bar{\eta }$ (-)	$Q_{\infty }^{e}$ (-)	$E_{a}/R$ (-)
w/c = 0.40	2.4	6.6	−0.046	1	11.5	108,700	5000

**Table 5:** Mix proportions of SSC concrete.

w/c	Composition of Concrete (kg/m^3^)							Set-Retarding Superplasticizer	Air Entraining Agent
	Water	Cement	Sand	Stone Small	Stone Middle	Stone Large	Stone Extra		
0.40	78	195	558	318	318	477	477	0.7%	0.02%
